# Cytokine Regulation of the Bone Pre- and Metastatic Niches: Implications for Breast Cancer Dormancy

**DOI:** 10.3390/cells15171528

**Published:** 2026-08-25

**Authors:** Tamara A. Clover, Maria L. Price, Lewis A. Quayle, Christine L. Le Maitre, Penelope D. Ottewell

**Affiliations:** 1 Division of Clinical Medicine, University of Sheffield, Beech Hill Road, Sheffield S10 2RX, UK; taclover1@sheffield.ac.uk (T.A.C.); m.l.price@sheffield.ac.uk (M.L.P.); c.lemaitre@sheffield.ac.uk (C.L.L.M.); 2 School of Biosciences and Chemistry, Sheffield Hallam University, Howard St, Sheffield S1 1WB, UK; l.quayle@shu.ac.uk

**Keywords:** breast cancer, bone metastasis, pre-metastatic niche, metastatic niche, dormancy, cytokine regulation

## Abstract

**Highlights:**

**What are the main findings?**

- Macrophage polarisation, CXCL9, 10 and 12 signalling support bone homing and dormancy of breast cancer. Cytokines LIF, TGF-β, BMP, FGF and interleukins maintain bone disseminated breast tumours in a dormant state.
- Within the bone, interactions between cancer cells and the metastatic niche disrupt the immune and cytokine landscape, permitting breast tumour re-awakening and subsequent metastatic outgrowth.

**What are the implications of the main findings?**

- Understanding cytokine-mediated breast tumour relapse in the bone has led to advancements in therapeutic intervention strategies, specifically targeting dormancy maintenance and eradication mechanisms.
- Novel therapeutic interventions are necessary for the targeting of dormant cells to prevent late-onset metastasis.

**Abstract:**

Breast cancer relapse in bone is a significant clinical problem that is experienced in ~70–80% of patients with late-stage breast cancer. This condition commonly occurs 5–10+ years following surgical removal of the primary tumour. The long latency seen prior to relapse in bone is a result of tumour cell dormancy. Once disseminated to the bone, tumour cell interaction with the bone metastatic niche (endosteal niche and endovascular cells) maintains cells in a dormant state until changes to the local environment activate the niche to support outgrowth. Amassing evidence suggests that cytokines are key regulators of the bone metastatic niche, controlling bone homing and metastatic outgrowth. Pro-inflammatory cytokines, including IL-1β, IL-6, IL-8, TGFβ and RANKL, play crucial roles in attracting tumour cells to bone. Furthermore, these cytokines act in conjunction with IFN, VEGF, TGF, PTHrP, FGF, OPG and various chemokines to regulate expansion of the niche, facilitating tumour cell escape from dormancy and promoting the “vicious cycle of bone metastasis”. Here, we review the current literature to provide an up-to-date understanding of how interactions between cytokine signalling cascades regulate the bone metastatic niches to promote homing, dormancy or metastatic outgrowth of breast cancers. Because breast cancers are predominantly osteolytic, this review focuses on dormancy and metastatic outgrowth associated with lytic disease in addition to current advances in novel therapeutics aimed at preventing this condition through targeting dormant cells.

## 1. Introduction

Female breast cancer is the second most common cancer diagnosed worldwide, with 666,000-reported deaths in 2022 [1]. Strikingly, the greatest cause of death in breast cancer patients is metastasis rather than the primary tumour itself [2], with 70–80% of late-stage breast cancer patients developing incurable bone metastases [3].

Within the bone, breast cancer cells have been found to home to the endosteal and bone marrow endovascular niche, where they can enter a prolonged state of dormancy [4,5]. Dissemination of breast cancer cells into this niche has been shown to occur early, before diagnosis of the primary tumour, with dormancy impeding their detection [5,6,7]. As a result, bone metastasis often occurs in patients 5–10+ years post diagnosis and after successful treatment (mastectomy, radiotherapy or breast conserving surgery) of the primary tumour [8]. Post mortem studies have shown that ~70% of breast cancer patients have disseminated tumour cells in their bone, suggesting that re-awakening of the dormant cells only occurs in a subset of breast cancer patients [9]. Because we are currently unable to identify which patients will experience dormant cell re-awakening and subsequent metastatic outgrowth, it is not possible to prioritise patients for preventative treatment/early detection strategies [10]. This current lack of biomarkers means bone metastases typically remain undiagnosed until patients present with skeletal-related events (SREs), including pain, fractures, compression of the spinal cord and hypercalcaemia [11]. Importantly, diagnosis of bone metastasis at this stage has a median survival of ~3 years [12], and studies of the bone metastatic niche indicate bone to represent a primary and transient site for metastatic breast cancer cells, whereby metastases develop in bone and/or bone acts as a reservoir for tumour cells seeding other organs [13,14]. Thus, a greater understanding of the bone metastatic niche and its regulation is necessary to improve early detection methods and novel therapies to reduce distal recurrence and improve survival in breast cancer. To initiate spread from the primary site, breast cancer cells disrupt normal homeostatic mechanisms in bone, priming the environment for their arrival [15]. Under normal physiological conditions, the bone marrow endosteal and endovascular niches support cell populations involved in the balance of bone production and degradation. These cell populations include osteoblasts and osteoclasts as well as osteocytes, haematopoietic stem cells (HSCs), mesenchymal stem cells (MSCs), adipocytes and various immune cell populations, including myeloid-derived suppressor cells (MDSCs), CD4^+^, CD8^+^ and T regulatory (Treg) cells [16]. Priming of the pre-metastatic niche (PMN) includes changes to the extracellular matrix (ECM), activity of resident stromal and immune cells, the cytokine/chemokine landscape, as well as the surrounding vasculature [15]. These processes support chemoattraction of circulating cancer cells, expansion of the metastatic niche and tumour cell survival [15]. The early concept of the PMN arose from Fuchs’ theory and supporting work by Paget that cancer metastasises to pre-determined sites—much like “seed and soil” [17].

Upon extravasation in the bone, tumour cells can locate to the metastatic niche, where tumour cell-niche cell interaction can influence cell fate [11]. Disseminated breast cancer cells can enter a cell-cycle arrested state, sometimes associated with a high p38:ERK ratio [18]. This quiescent state is referred to as dormancy, and although non-proliferative, these cells remain transcriptionally active [19]. The two subtypes of dormancy include tumour-mass dormancy or cellular dormancy [19,20], the latter aligning most with the classical definition of dormancy [19]. In the context of breast cancer (the focus of this review), cellular dormancy refers to a non-proliferative yet metabolically active state of breast cancer cells, induced via repressive microenvironment-derived signalling [10,19]. This quiescence is inferred to serve as a protective state for disseminated tumour cells, aiding tumour survival at distal regions as well as withstanding therapeutic interventions [19]. Alternatively, tumour-mass dormancy refers to a state of equilibrium between cancer cell death and proliferation, on a tumour-wide basis [10,20] and therefore net zero growth of the tumour [19]. Tumour-mass dormancy is regulated by the microenvironment through cancer-stromal cell interactions, angiogenesis and immunosurveillance [10]: Angiogenic dormancy is induced via secretion of anti-angiogenic factors and inhibition of pro-angiogenic factors. Subsequently, an angiogenic switch occurs, promoting angiogenesis and eliciting tumour outgrowth. Whereas immunologic dormancy refers to the immune clearance of subpopulations of dormant cells and immune escape of others—once again resulting in an equilibrium between cancer cell persistence or exclusion from the niche [10]. The microenvironment in which dormant disseminated cancer cells reside is referred to as the metastatic niche and this niche is responsible for the maintenance of dormancy prior to tumour outgrowth. Importantly, the metastatic niche is regulated by various families of cytokines [4,21], and it is suggested that both types of dormancy (cellular and tumour-mass) may exist simultaneously within the same niche—potentially causing further difficulty in the detection of dormant cells prior to tumour outgrowth [10].

Upon dormant tumour cell re-awakening, one characteristic of breast cancer bone metastasis is abnormalities in osteoclast and osteoblast factor secretion, leading to lytic or sclerotic lesion formation, respectively [22]. Importantly, the majority of breast cancer bone metastases are classified as osteolytic, and hence the mechanisms outlined in this review are focused on the drivers of lytic disease [23]. Metastatic breast tumour growth is sustained and amplified through a positive feedback loop, “the vicious cycle of bone metastases”. Briefly, this cycle is driven by growth factor release from the extracellular matrix (ECM), as a result of cancer cell activity promoting heightened osteoclast-mediated resorption [22]. The resulting increase in growth factors within the niche promotes tumour growth, which further supports bone resorption and the cycle is repeated [22]. This review aims to elucidate our current understanding of the cytokine networks that regulate breast cancer bone pre-metastatic and metastatic niches, specifically focusing on the regulation of breast cancer dormancy and reactivation.

## 2. The Homeostatic Bone Niche

Within the bone marrow and endosteal niche, osteoblasts and osteoclasts perform opposing functions, maintaining homeostasis of bone and skeletal tissue. Zones of micro-injury are resorbed and replaced with newly synthesised bone, allowing for consistent mechanical stress tolerance [16,24].

**Figure 1:**
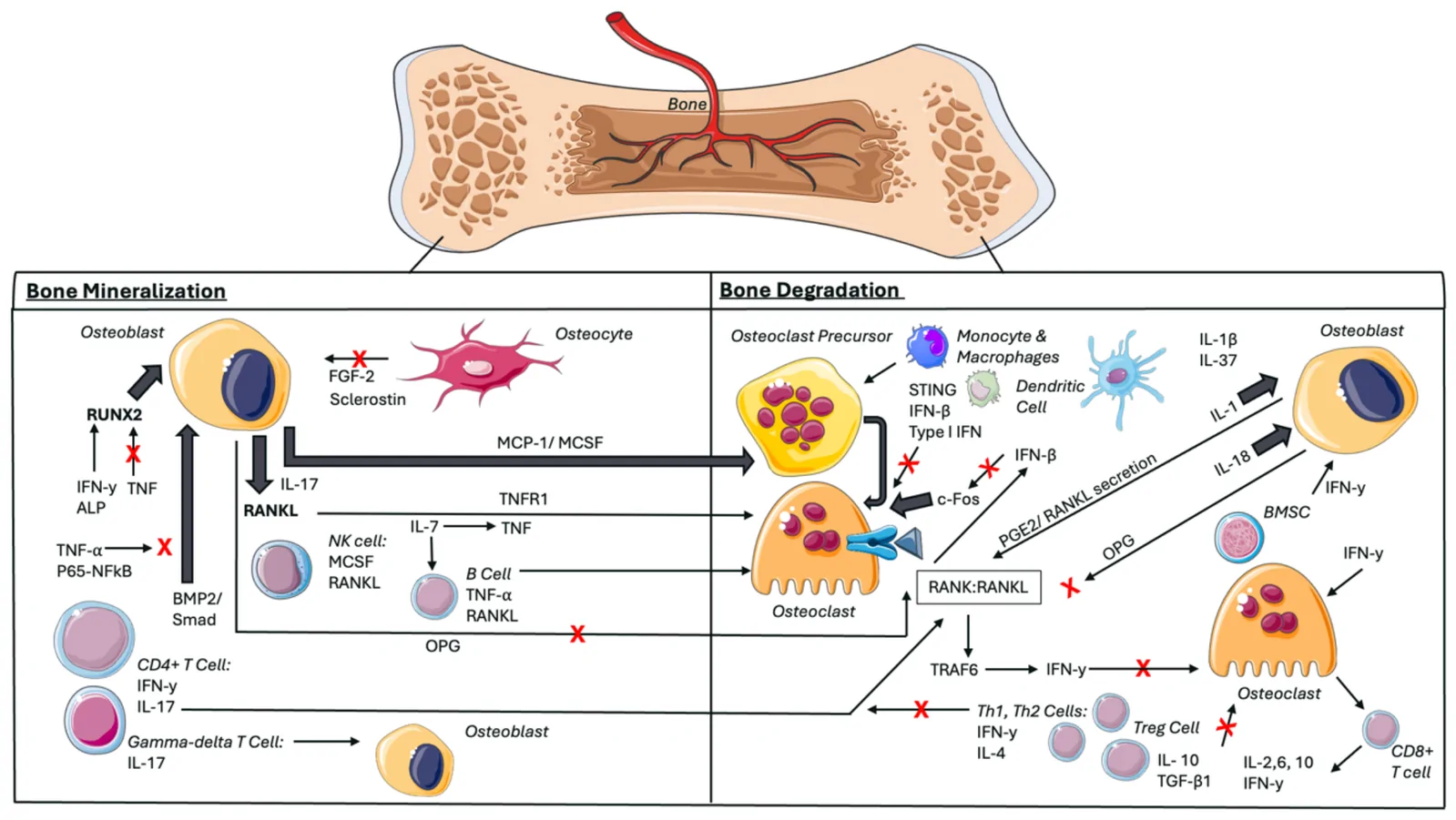
Cytokine and immune cell regulation of the bone homeostatic niche.

Osteoblasts synthesise and secrete new matrix (mineralisation) to replace micro-injured bone, before maturing into terminally differentiated osteocytes, embedded in the matrix as mechano-sensors [24,25]. Upon injury detection, osteocytes regulate concentrations of osteoblasts and osteoclasts via cell–cell contact or secretion of soluble factors [24]. These include sclerostin and fibroblast growth factor 2 (FGF-2), which reduce bone mineralisation [25]. Osteoblasts secrete monocyte chemoattractant protein 1 (MCP-1) and macrophage colony-stimulating factor (MCSF), driving the recruitment of osteoclast precursors and the secretion of key cytokines such as receptor activator of NFκB ligand (RANKL), further favouring osteoclast-mediated bone resorption (Figure 1) [24]. Osteoblast-secreted osteoprotegerin (OPG) is a competitive inhibitor of RANKL, blocking the binding of RANKL to its receptor (RANK) and impeding osteoclast maturation, supporting bone homeostasis when appropriate [26]. For example, it has been suggested that osteoblasts in early developmental stages, as well as osteocytes, secrete RANKL, but in more mature osteoblasts, OPG secretion is favoured [26].

Regulation of the bone homeostatic niche is, in part, driven by osteoblast- and osteoclast-derived cytokine secretions. Recruitment of osteoclast precursors to the bone niche is mediated by osteoblast secretion of both monocyte chemoattractant protein 1 (MCP-1) and macrophage colony-stimulating factor (MCSF) [24]. Osteoblast secretion of receptor activator of NFκB ligand (RANKL) promotes osteoclast maturation and bone resorption upon binding to RANKL receptor (RANK) [24,26]. Osteoblast-derived osteoprotegerin (OPG) competitively inhibits this receptor-ligand interaction, impeding osteoclast-mediated bone resorption when necessary [26]. Tumour necrosis factor (TNF), including TNF receptor 1 (TNFR1) signalling, has been found to upregulate RANKL and, synergistically, amplify osteoclast-mediated resorption, whilst inhibiting osteoblast differentiation via blocking of runt-related transcription factor 2 (RUNX2) DNA binding [27,28,29,30]. In collaboration with NFκB, TNF further blocks osteoblast differentiation via the inhibition of bone morphogenetic protein 2 (BMP2)/Smad signalling [31]. The interleukin (IL) family displays varying effects regarding regulation of bone homeostasis. IL-1 has been found to increase secretion of RANKL, promoting osteoclast-mediated bone resorption. IL-1 also demonstrates direct effects on the proliferation of osteoblasts when added to cultures of OB1 cells [32,33]. In contrast, a member of the IL-1 family, IL-37, has been shown to inhibit osteoclast-mediated resorption [34]. IL-37, however, also inhibits IL-18 during IL-1β derived inflammation [34]. IL-18 stimulates OPG production and the inhibition of osteoclast-mediated resorption, therefore highlighting the dual role of IL-37 and the environment-dependent regulation of bone homeostasis [35]. Further inhibition of osteoclast differentiation and bone resorption is attributed to the interferon family (IF), namely IFNβ, and IFN-γ [36]. Importantly, IFN-γ also promotes osteoclast-mediated bone resorption; for example, via osteoclast differentiation of macrophages [37]. Osteoblast-specific effects include an IFN-γ-driven increase in alkaline phosphatase (ALP)—resulting in upregulation of Runx2 and therefore also osteoblast-dependent bone matrix mineralisation [38,39]. Image(s) provided by Servier Medical Art (https://smart.servier.com), licensed under CC BY 4.0 (https://creativecommons.org/licenses/by/4.0/) (Accessed on 23 August 2026).

### 2.1. Cytokine Regulation of the Homeostatic Bone Environment

In addition to the classically defined RANKL-OPG regulation of bone homeostasis, tumour necrosis factor (TNF), interleukins (IL), and the interferon (IFN) family of cytokines have also been identified to play crucial roles (Figure 1) [27]. TNF has been shown to work in synergy with RANKL, eliciting pro-osteoclastic and anti-osteoblastic functions, specifically in TNF excess states [28,29]. Signalling through TNF receptor 1 (TNFR1) was found to upregulate RANKL and stimulate subsequent osteoclast differentiation [29]. TNFR1 has also been implicated in the inhibition of osteoblast differentiation, via TNF-driven blocking of the runt-related transcription factor 2 (RUNX2) DNA binding [30]. RUNX2 is named “the master regulator of osteoblastogenesis” and, in accordance, blocking RUNX2 activity inhibits this [30]. Furthermore, TNF has been proposed to interfere with nuclear bone morphogenetic proteins (BMP)/Smad signalling, and BMP-2-driven osteoblast differentiation was found to be inhibited by TNF, specifically in collaboration with NFκB signalling via the NFκB p65 subunit [31].

Like TNF, the IL family also displays pro-osteoclastic characteristics, as well as several regulatory effects on the bone environment. The IL-1 family, in particular, has been shown to stimulate prostaglandin E2 and RANKL secretion from osteoblasts, supporting osteoclastogenesis [32]. In contrast, IL-18 was found to counteract this by stimulating OPG production from osteoblasts, competitively inhibiting osteoclastogenesis [35]. This was more recently supported by mouse model studies, whereby mice genetically deficient in IL-1 demonstrated increased bone mass [32], and transgenic mouse models with amplified IL-18 displayed reduced bone turnover [40]. Furthermore, IL-37 was recently identified as a member of the IL-1 family and found to inhibit osteoclast differentiation [34]. It has been demonstrated that a pro-inflammatory environment, namely IL-1β-derived inflammation, stimulates the secretion of IL-37, which is also a naturally occurring inhibitor of the pro-inflammatory cytokine IL-18 [34]. Additional evidence for IL-1β in bone homeostasis has arisen from in vitro studies, whereby additional supplementation of exogenous IL-1β to cultures of OB1 osteoblast cells led to an increase in OB1 proliferation [33]. This highlights a specific role for IL-1β in bone homeostasis, through direct effects on resident cell populations, but also the complex overlaps between cytokine networks involved in regulation of the bone environment.

This complexity is reinforced through further inhibition of osteoclastogenesis by the IFN family of cytokines. Activation by RANKL has been shown to induce cellular secretion of IFNβ, which in turn inhibits the transcription factor for osteoclast differentiation—c-Fos (Figure 1) [36]. Osteoclastogenesis was additionally found to be hindered by IFN-γ inhibition of TNF receptor-associated factor 6 (TRAF6) signalling, following RANK–RANKL engagement [36]. However, it is important to highlight that depending on the inflammatory status, IFN-γ has been found to also elicit pro-resorptive characteristics [37]. Evidence suggests that IFN-γ-secreting Th1 cells can directly stimulate RANKL-dependent osteoclast differentiation of macrophages; however, this is not the case for non-IFN-γ-secreting T cells [37]. Furthermore, stimulator of interferon genes (STING) stimulation of type I IFN transcription has been suggested to contribute to bone homeostasis, supported by previous studies displaying STING activation to impede osteoclast activity—specifically through IFNβ, and type I *IFN* gene stimulation [41]. Type I IFNs act on the signal transducer and activator of transcription 1/2 (STAT1/STAT2) and interferon regulatory factor 9 (IRF9), via their receptor IFNAR1/2, resulting in increased expression of the interferon-stimulating gene family [36]. IFN-γ has also been found to affect osteoblasts by increasing alkaline phosphatase (ALP) activity in human femur. This upregulates the transcription factor Runx2, and therefore matrix mineralisation by osteoblasts [38,39], further evidencing the environment-dependent dual role of IFN with regards to bone remodelling [36,37,38,39,41].

### 2.2. Physiological Osteoimmunology of Bone Homeostasis

The bone is a haematopoietic environment, where various immune cell populations, including dendritic cells, T cells (CD4^+^, CD8^+^ and Treg), B cells and NK cells, reside [39]. Within the bone, local monocytes and macrophages can differentiate into osteoclasts, contributing to bone resorption and degradation [42]. Similarly, bone marrow mesenchymal stem cells (BMSCs) differentiate into osteoblasts, counteracting the resorptive activity of osteoclasts and promoting matrix mineralisation/bone formation [39,43].

Here, osteoimmunology will be discussed particularly in relation to the crosstalk between immune cell types and bone-resident osteoclasts and osteoblasts. This is highly relevant due to skeletal and immune systems sharing overlapping RANK/OPG networks, as evidenced in RANKL-deficient mice exhibiting both severe osteoporosis and defects in the development of T and B cells intrinsic to the bone [44].

#### 2.2.1. NK Cells

Importantly, immune cells in the bone environment have been identified as key sources of cytokines involved in the regulation of bone remodelling and the overall bone niche [45]. With regard to osteoclastogenesis, RANKL and MCSF are key cytokines involved in the process of osteoclast differentiation, whilst also being expressed on the surface of NK cells [45]. Co-culture studies between monocytes and NK cells harvested from a rheumatoid arthritis synovium were found to stimulate osteoclast differentiation in vitro [45]. Furthermore, NK cell depletion in mouse models prior to the induction of arthritis led to almost complete impediment of bone erosion, as well as reduced severity of the arthritis [45]. Therefore, NK cells harbour a potential role in osteoclastogenesis, in line with their expression of key cytokines [45].

#### 2.2.2. T Cells

On the other hand, activated CD4^+^ T cells have been found to promote MSC differentiation into osteoblasts, as well as CD4^+^ IFN-γ secretion, inhibiting MSC differentiation into adipocytes [39]. Furthermore, MSC’s harvested from IFN-γ receptor 1 (IFN-γR1) knockout (KO) mice (IFN-γR1 −/−) are less capable of differentiating into osteoblasts, and the mice themselves exhibit reduced bone mineral density [46], highlighting a role for CD4^+^ derived IFN-γ secretion in osteoblastogenesis within the bone.

In continuation with CD4^+^ lineage cells, CD4^+^ Th17 cells have been previously identified as a facilitator of T cell-driven osteoclastic bone resorption in autoimmune arthritis models [47]. In an osteoclast differentiation co-culture system (established through culturing bone marrow cells with Th17 cells and osteoblasts, supplemented with Vitamin D3 (1,25-(OH)_2_) and Prostaglandin E2), high amounts of IL-17A were detected, as well as increased osteoclastogenesis markers [47]. Interestingly, when Th17 cells were isolated from IL-17 −/− mice, osteoclastogenic effects were hindered. Furthermore, the same was observed when Th17 cells were cultured in the absence of osteoblasts, but in a RANKL/MCSF culture system. This suggests that Th17 cells indirectly promote osteoclastogenesis, potentially via IL-17A-driven RANKL secretion from osteoblasts [47]. Furthermore, using the above co-culture model, Th1 and Th2 cells were found to secrete IFN-γ and IL-4, respectively [47], and as a result, Th17-driven osteoclastogenesis was impeded, and the development of osteoclast precursors into mature successors inhibited [47]. This is important as it highlights the differing roles of immune cells in maintaining bone homeostasis, in this case, via the promotion or impeding of osteoclastogenesis.

In contrast to CD4^+^ Th17 cells, CD8^+^ T cells (specifically FOXP3-expressing) have been shown to inhibit osteoclast-mediated bone resorption via secretion of IL-2, 6, 10 and IFN-γ cytokines [48]. Interestingly, this observation was made following their activation by osteoclasts themselves [48]. Additional evidence for the role of IFN-γ in osteoclastogenesis was uncovered from the culturing of human monocytes with MCSF [49]. This led to inhibited osteoclastogenesis upon addition of RANKL with IFN-γ, and was suggested to be due to IFN-γ cooperation with toll-like-receptor (TLR)—reducing RANK expression [49]. Collectively, this evidence reinforces immune cell-driven regulation of osteoclastogenesis, but also proposes regulation to be through an osteoclast-mediated, immune cell-driven, negative feedback loop [48,49].

Furthermore, during the development and survival of CD8^+^ memory T cells, the Janus kinase (JAK)/STAT and phosphoinositide 3-kinase/protein-kinase-B (PI3K/Akt) pathways are stimulated via stromal cell-secreted IL-7, following activation by stromal cell-derived IL-1β and TNF-α [50]. It was found that secretion of IL-7 also further amplified the T cell production of TNF-α and RANKL, driving bone loss in vivo and linking the cytokine to greater levels of osteoclastogenesis and bone resorption [50]. Interestingly, macrophages from the synovial fluid of rheumatoid arthritis patients were found to have high levels of IL-7 and its receptor [51].

#### 2.2.3. B Cells

IL-7 has also been identified in B cell lymphopoiesis [52], and with regard to bone resorption and matrix mineralisation, B cells have exhibited dual roles, identified to mediate osteoclast-driven bone resorption via TNF secretion, yet also impede osteoclastogenesis via transforming growth factor beta (TGF-β) secretion [53,54]. Importantly, B cells in the bone marrow have also been identified as key sources of OPG—hindering RANKL-dependent osteolysis [55].

#### 2.2.4. Other T Cell Subsets

Lastly, additional immune cell subtypes which have displayed key roles in the regulation of the skeletal environment include Treg and gamma-delta T cells. Treg cell secretion of IL-10 and TGF-β1 (TGF-β isoform 1) has been associated with hindered osteoclast differentiation and therefore bone resorption [56], whereas gamma-delta T cells’ secretion of IL-17A has been proposed to promote bone formation [57]. Furthermore, the inflammatory environment of the bone and prevalence of cytokines IL-1β, 6, 17, TNF, and RANKL have been found to establish an environment supportive of dendritic cell differentiation into osteoclasts [27,58], highlighting a more complex crosstalk between the immune and skeletal systems.

Cumulatively, the above findings bridge the gap between the bone and immune system. These findings highlight inter- and intra-family cytokine interactions, as well as immune networks, in maintaining bone homeostasis. Changes to these cytokine and immune cell balances contribute to the formation of various niches in the bone. The following sections will focus on the bone pre-metastatic and metastatic niches, and their roles in the dormancy and outgrowth of disseminated breast cancers.

### 2.3. Pathological Tumour Osteoimmunology

In contrast to physiological osteoimmunology, breast cancer relapse in the bone results in re-composition of osteoimmunology to support tumour survival [59], herein referred to as pathological tumour osteoimmunology.

#### 2.3.1. Neutrophils, T Cells and NK Cells

During breast cancer metastasis, it has been shown that IL-1β secreted at the primary tumour site causes increased secretion of IL-17 from gamma-delta T cells, and phenotypic alteration of neutrophils to favour iNOS inhibition of CD8^+^ (anti-tumour) T cells. Cumulatively, this enhances the ability for tumour cells to circulate and seed at distant sites, establishing overt metastases [60]. This has been specifically demonstrated in mouse models of breast cancer lung metastases [60]. However, it has been speculated that this surge of inflammation at the primary site causes retreat of immune cells from the bone marrow [61]. Reductions in CD4^+^ and CD8^+^ cytotoxic T cells, as well as reductions in NK cells, have been observed in breast cancer-specific models using an in vivo mouse model of spontaneous breast cancer (triple negative, 4T1, breast cancer cells) [59]. Furthermore, it has also been shown that microenvironment-derived IL-1β increases infiltration of anti-tumour neutrophils at the primary breast tumour site, but the opposite is found in the bone [61]. This is important as it demonstrates the varying composition of the immune landscape, dependent on the site of breast cancer metastasis, but also the tumour-favouring characteristics of the bone marrow.

#### 2.3.2. Tumour Associated Macrophages

Macrophage polarisation regulates breast cancer dormancy or outgrowth in the bone, with M1-like macrophages supporting tumour proliferation and M2-like macrophages supporting tumour dormancy [62]. Within the bone niche, macrophages polarised to the tumour-associated macrophage (TAM) phenotype are associated with poor cellular cytotoxicity and immune suppression [63,64]. More specifically, the mechano-exclusion of CD8^+^ T cells from the TME, via TAM-mediated collagen VIA/XII deposition and stiffening of the TME, has been demonstrated by Tharp et al. in PyMT mouse models of breast cancer [63]. Furthermore, using a spontaneous, in vivo, model of breast cancer (KEP transgenic mouse), TAMs were observed to further suppress the immune response of the TME via accumulation of T regulatory cells [64].

Lastly, information obtained from in vivo mouse models have also shown that exosomes derived from 4T1 breast cancer cells enhance the secretion of IL-1β, IL-6, and TNF-α from TAMs [65]. This is important as microenvironment-derived IL-1β has been associated with the progression of disseminated breast cancers and bone metastasis [61], reinforcing the role of TAMS in promoting the progression of breast tumours within the bone metastatic niche.

#### 2.3.3. Fibroblasts

Metastatic breast cancer cells, having exited dormancy, upregulate the production of osteoblast-secreted inflammatory cytokines [66]. This surge in inflammation supports quiescent fibroblasts’ transition to a cancer-associated phenotype, secreting factors to promote primary tumour growth, as well as potentially the outgrowth of metastatic breast tumours in the bone [66]. In triple-negative breast cancer, it was found that tumour-derived IL-1β, in a hypoxic environment, promotes CAFs to express a phenotype with greater invasive characteristics. This was measured using a Transwell invasion assay, exposing CAFs to media collected from MDA-MB-231 breast cancer cells exposed to hypoxic conditions for 16 h [67]. Importantly, CD105^+^/CD34^−^ CAFs have been associated with a greater rate of occurrence of bone metastasis in breast cancer patients [68].

#### 2.3.4. B Cells

Lastly, during metastatic outgrowth of breast cancers, the osteoimmunological secretome of the bone shifts to support the vicious cycle of tumour growth. For example, in breast cancer bone metastasis, specifically, B cells have been suggested to undergo a secretome shift, increasing the production of RANKL over OPG and amplifying osteolysis via increasing osteoclastogenesis [55]. Cumulatively, this highlights the role of breast cancer colonisation in remodelling the immune landscape of the bone to favour tumour survival.

## 3. Priming of the Bone Niche and Breast Cancer Homing

### 3.1. The Pre-Metastatic Niche (PMN)

Disseminated breast cancer cells reside in the bone niche prior to diagnosis of clinically detectable overt metastasis [69,70]. For example, Braun et al. identified 36% of a stage 1–3 breast cancer patient cohort (*n* = 552) to have breast cancer cells present in the bone marrow at the time of primary tumour surgical resection [69]. During a 4-year follow-up period, it was reported that these patients, with micro-metastasis in the bone, were associated with subsequent detection of overt metastatic lesions or death from cancer-related causes (24.6%) (*p* < 0.001) [69]. Supporting evidence from Hüsemann et al. also demonstrated that bone marrow disseminated cancer cells spread during the early stages of breast cancer, specifically during atypical ductal hyperplasia [70]. Collectively, the above literature supports the notion of PMN priming by the primary tumour to support early dissemination of cancer cells, and dormancy aiding cancer survival, until a permissive environment supports tumour growth/overt metastasis [4,15,21].

The concept of tumour cell priming of a PMN, and the involvement of cytokine signalling, is evidenced in work carried out by Kaplan et al. [15]. The group’s work demonstrated the role of vascular endothelial growth factor receptor (VEGFR)-expressing bone marrow-derived cells (BMDCs) in pre-metastatic homing and clustering, with inhibition of VEGFR inhibiting metastasis of Lewis Lung Carcinoma (LLC). The greatest BMDC clustering was identified in the lungs and liver of mice following injection with LLC cells; however, VEGFR1 clustering was also observed in the axillary lymph node prior to the dissemination of breast carcinoma cells [15].

**Figure 2:**
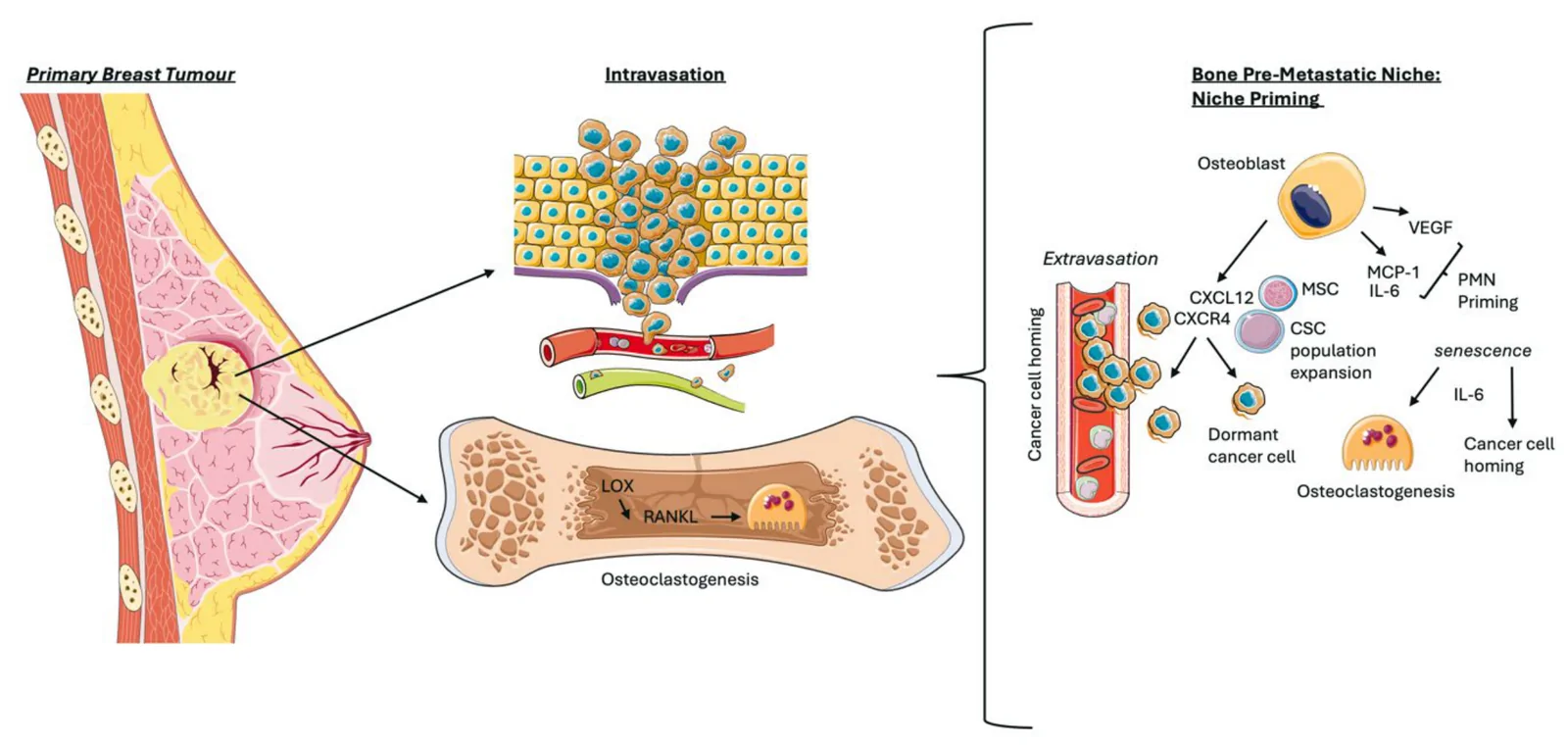
Tumour priming of the breast cancer bone pre-metastatic niche, tumour cell dissemination and homing.

Later studies also identified osteoblast-derived VEGF in the metaphysis of the trabecular bone in breast cancer models [71]. These findings were coupled with cytokine(s) MCP-1 also residing in the metaphysis, and IL-6 in the marrow [71]. Collectively, these findings suggest that VEGF, MCP-1 and IL-6 cytokines facilitate “priming” of the bone as a site for circulating breast cancer cell seeding [15,71]. Interestingly, IL-6 is a senescence-associated secretory phenotype (SASP) factor [72] and its inhibition via neutralising antibody treatment reduced tumour cell homing to the bone, but also senescence-related osteoclastogenesis [72]. Moreover, RANKL-driven osteoclastogenesis is amplified following lysyl-oxidase (LOX) secretion from the primary tumour [73]. Once again, this supports the notion that primary tumour-secreted factors prime distant sites for the arrival of circulating cancer cells, prior to the formation of overt metastases (Figure 2).

Osteoblast secretion of the cytokines vascular endothelial growth factor (VEGF), monocyte chemoattractant protein 1 (MCP1) and interleukin 6 (IL-6) has been suggested to facilitate the distant “priming” of the bone pre-metastatic niche (PMN)—or preparation of bone for tumour cell arrival [15,71]. In addition, secretion of lysyl-oxidase (LOX) from the primary tumour is suggested to precede increased levels of RANKL-dependent osteoclast-mediated bone resorption, prior to metastasis of the cancer [73]. Recruitment of cancer cells to the bone niche is via chemoattraction between bone stromal-derived CXCL12 ligand and cancer cell surface expression of the CXCR4 receptor [74,75]. Cancer cell entry into the bone has also been suggested to be amplified via mesenchymal stem cell (MSC) interaction with surface CXCR4, and this interaction is also suggested to increase the cancer stem cell (CSC) populations within the bone, creating a niche permissive for cancer survival [76,77]. Images provided by Servier Medical Art (https://smart.servier.com), licensed under CC BY 4.0 (https://creativecommons.org/licenses/by/4.0/) (Accessed on 23 August 2026).

In both studies exploring PMN priming by the primary tumour, it is important to acknowledge that in the clinic, dissemination of breast cancer cells to the bone has been reported to occur prior to diagnosis of the primary tumour [5,6,7]. This raises the question as to whether or not observations of PMN priming in mice are clinically translatable to human breast cancers, whereby during dissemination to the bone, primary tumour secreted factors may not be of high enough abundance to elicit the same effects as observed in mice—due to the small size of tumours at this stage, and their lack of clinical detection.

In this case, it could be argued that the intra-cardiac injection of cancer cells, as seen in mouse models from Bussard et al. [71], may amplify the clinical relevance of experimental observations. Intra-cardiac injection bypasses the early stages of the metastatic cascade, and tumour cells are guided, from the bloodstream, by homing factors to their preferred sites of metastasis [78]. This may eliminate the need for PMN priming prior to bone metastasis and therefore, in the context of clinical breast cancers, the experimental observations may be more relevant. It would be interesting to replicate the findings of Kaplan et al. [15] using intra-cardiac injection of breast cancer cells to emulate a more clinically relevant model. Similarly, it could be argued that the bone harbours a natural competency for disseminated breast cancer cell seeding, regardless of the PMN. For example, due to the CXCL12-CXCR4 axis, it may be argued that breast cancer cells are naturally wired to disseminate to the bone, as well as bone marrow expressing adhesion molecules, such as osteopontin or CD44, promoting breast cancer cell seeding to the niche [74,75,79]. However, these discussions remain in their infancy and do not account for the role of the PMN in promoting and maintaining dormancy of these disseminated cancer cells—as outlined in this review.

### 3.2. Cytokine-Mediated Homing of Circulating Breast Cancer Cells to the Bone Pre-Metastatic Niche

Cancer cells utilise the CXCL12-CXCR4 chemokine axis to promote metastasis, intravasation and lodging of cancer cells in the metastatic niche of the bone (Figure 2) [74,75]. CXCL12 drives increased vascular permeability, and therefore the exit of cancer cells from the vasculature into the surrounding tissues [75]. CXCL12 is secreted by bone-resident osteoblasts [66], aiding the chemoattraction and homing of CXCR4-expressing cancer cells to the bone niche [66]. When breast cancers come in contact with the stroma, it has been suggested that miRNA-driven downregulation of CXCL12 takes place as a result of gap junctional intercellular signalling. This reduction was found to correlate with reduced proliferation of the breast cancer cells in the bone, in support of dormancy [80]. Furthermore, Corcoran et al. suggested that breast cancer cells interact with MSCs via the CXCR4 receptor, supporting their entry into the bone [76]. Importantly, co-culture studies between breast cancer cells and bone marrow-derived MSCs resulted in increases in cancer stem cell population, measured through CD24^−^and CD44^+^ marker expression [77]. These findings support CXCL12/CXCR4-expression in the early stages, being deemed a pro-dormancy factor promoting niche colonisation and cancer survival. This is reinforced by Müller et al., who conducted mRNA quantification of homogenised tissue samples, uncovering the highest levels of CXCL12 expression to be in organs correlating with the most common metastatic sites of breast cancer, including the bone [81]. Additionally, CXCR4 signalling was found to drive pseudopodia formation in breast cancer cells, mediating cancer cell invasion and reinforcing the role of the CXCL12–CXCR4 axis during breast cancer invasion of the bone [81].

On the other hand, Sun et al. [82] observed a seemingly “dual” role for CXCL12/CXCR4 in breast cancer progression. MDA-MB-231 breast cancer cells were stably transfected with CXCL12, followed by a Matrigel invasion assay 48 h and 72 h later. Transfected cells experienced an increase in their invasive capabilities, reduced proliferation or apoptosis [82]. A suggested explanation for this confounding observation, considering findings from the group’s previous work, is that cell growth was favoured for malignant cells with greater invasive capabilities and apoptosis favoured for the less-invasive cell populations [82]. However, this hypothesis remains to be validated and uncovers a more complex role for CXCL12/CXCR4 in breast cancer metastasis.

## 4. Cytokine Regulation of Breast Cancer Dormancy in Bone

Upon cancer cell seeding at the metastatic site, a variety of additional cytokines have been identified to aid the regulation of cancer dormancy within the bone niche. These include leukaemia inhibitory factor (LIF), CXCL9 and CXCL10, TGF-β and FGF-2 (Table 1) [83,84,85,86,87,88]. Here, these cytokines will be discussed regarding their roles across proliferative, immune-mediated and angiogenic dormancy of breast cancers in the bone.

Both dormancy and outgrowth of breast cancers in the bone are regulated by various families of cytokines. Key cytokines during dormancy maintenance include leukaemia inhibitory factor (LIF), CXCL9, 10, transforming growth factor beta (TGF-β) and fibroblast growth factor 2 (FGF-2) [83,84,85,86,87,88]. Downregulation of the LIF receptor has previously been suggested to support outgrowth of dormant cancer cells in the bone—driven by hypoxia [83]. CXCL9 is suggested to potentially support dormant breast cancer outgrowth in the bone via anti-angiogenic characteristics, specifically by blocking VEGF binding to the endothelial cell surface [84,89,90]. CXCL10, more recently, was uncovered to potentially regulate immune-mediated dormancy of breast cancers in the lung metastatic niche via the CXCR3 receptor [86]. Additional experimental evidence is necessary to explore this function in relation to dormant breast cancer cells of the bone metastatic niche. In contrast, TGF-β has displayed dual functions regarding dormancy. In favour of dormancy, hypoxia drives TGF-β2 secretion by MSCs in bone and TGFβRIII downstream SMAD2 signalling/p27 expression to support growth arrest [91,92]. Furthermore, a synergy between TGFβ2 and BMP4 was found to hinder proliferation and induce/maintain dormancy in breast cancer cells in vitro [93]. In addition, FGF-2 co-signalling with α5β1 integrin has also been shown to drive dormancy of breast cancer cells cultured in vitro on fibronectin [88]. Immune-mediated dormancy of breast cancer cells in the bone is largely attributed to inflammatory monocytes and M2-like macrophages. Inflammatory monocytes, as well as tumour-derived vascular endothelial growth factor (VEGF), are suggested to increase infiltration of myeloid-derived suppressor cells (MDSCs) to the bone niche, promoting immune evasion of dormant cancer cells [94,95]. Dormant cancer cells secrete cytokines including interleukin 10 (IL-10) and TGF-β which support immune evasion of breast cancer cells through reduction in T and NK cell activity [91]. On the other hand, M1-like macrophages have been associated with supporting anti-tumour immune responses [96,97]. This is partially mediated by the secretion of pro-inflammatory cytokines: IL 1,2,6 and 12 as well as tumour necrosis factor (TNF) and chemokines CXCL9,10,11 [96,97]. Further activation of dormant breast cancer cells is because of osteoblast and tumour-derived cytokine secretions. High IL-1β in the primary tumour of patients has been associated with increased relapse of breast cancer in the bone [3,7,33,98]. Additional interleukins include IL-6,8 and 11. Signalling of these interleukins, as well as parathyroid hormone-related protein (PTHrP), VEGF, tumour necrosis factor alpha (TNF), receptor activator of NFκB ligand (RANKL), and TGF-β, support the vicious cycle of breast cancer bone metastasis [99,100]. Osteoprotegerin (OPG) is a decoy receptor for RANKL and is therefore implicated in the dormancy of breast cancers in the bone [101,102]. More recent data have suggested that immune-derived cytokines driving the outgrowth of breast cancers include Th17-derived IL-17A and IL-22. IL-17A. These cytokines have been found to support VEGF and matrix metalloproteinase (MMP)-driven degradation of the extracellular matrix, as well as stimulation of the IL-6,8, and 1β secretion [103,104]. Collectively, this reinforces the vicious cycle of breast cancer bone metastasis. Similarly, IL-22 was found to drive MMP-9 expression, specifically in relation to the activation of TGF-β and lysyl-oxidase (LOX), supporting bone homing and activation of breast cancer cells [105,106]. Lastly, chemokine ligand CXCL5 and receptor CXCR2 have been identified as potential regulators of invasion and angiogenesis of breast cancers in the bone, as well as CCL5 associated with the dormancy exit of breast cancers in the bone [107,108].

### 4.1. Proliferative Dormancy

LIF is a member of the interleukin cytokine family and is involved in supporting quiescence of breast cancers in the bone [83]. Downregulation of LIF receptor (LIFR), and subsequently downstream STAT3/SOCS3 signalling, supported the outgrowth of dormant cancer cells into metastatic lesions of the bone [83]. Moreover, patients predicted to experience recurrence in the bone, using the Van’t Veer signature, were found to present with low levels of LIFR mRNA within their primary tumours [83]. Interestingly, hypoxia is a key regulator of LIFR, reducing LIFR:STAT3 signalling and supporting breast cancer outgrowth in the bone [83].

Furthermore, it was found that hypoxia also induced elevations in several markers, including TGF-β [91]. TGF-β has been associated with a dual role regarding the dormancy and outgrowth of tumours in the bone. TGF-β resides in the ECM, and during dormancy its canonical signalling has been linked with the upregulation of growth arrest genes. However, activation of its non-canonical signalling pathway can promote tumour outgrowth and proliferation [87]. It could be hypothesised that upon initial cancer cell seeding, ECM remodelling drives TGF-β release to promote dormancy of seeding cells. However, upon appropriate environmental stimulation, TGF-β then begins signalling in a non-canonical manner, supporting tumour outgrowth over dormancy in this case [87].

TGF-β2 (TGF-β isoform 2) is secreted by Nestin+ MSCs in the bone marrow, maintaining dormancy of disseminated cancer cells residing in the bone marrow niche. This mechanism is hypothesised to be via the TGFβRIII receptor and downstream SMAD2-related upregulation of p27 [92]. In breast cancer specifically, TGF-β2 has been implicated in dormancy mechanisms within the bone microenvironment, acting in synergy with bone morphogenetic proteins (BMP) [93]. BMP4 was previously shown to display widespread expression in 21 out of 22 breast cancer cell lines, as part of a comprehensive study exploring BMP expression in breast cancer [109]. More recently, in the doctoral thesis of Risson (2022) [93], BMP4 and TGF-β2 were found to hinder proliferation of murine PYMT breast cancer cells in a 3D Matrigel model, as well as co-inducing/maintaining dormancy of breast cancer cells—uncovered through FUCCI cell sorting and pathway enrichment analysis [93]. Risson (2022) also uncovered that aged human bone marrow harbours lower levels of BMP4, highlighting a potential mechanism by which dormant breast cancer cells may re-awaken later in the lifetime of patients [93].

Lastly, FGF-2 was found to be abundant in bone marrow and deposited on ECM, and its loss was implicated in the malignant transformation of MCF-7 breast cancer cells in 3D culture [88]. Tivari et al. demonstrated that FGF-2 induced a dormant state in these cells, specifically when cultured on a fibronectin substratum [88]. In this model, the group found that although FGF-2 alone could not induce dormancy, when ER+ cells were co-cultured with FGF-2 on fibronectin, dormancy was observed [88]. The group also discovered that α5β1 signalling was required in parallel to FGF-2 to drive this dormancy [88]. In addition, FGF-2 upregulated inhibitors of cell-cycle-progressing cyclin-dependent kinases. This resulted in increased G1 growth arrest, leading to activation of ERK and PI3K, and increased expression of the α5β1 receptor [88]. Signalling through both receptors activated focal-adhesion-kinase (FAK) and the RhoA GTPase-activating-protein (GAP)/GTPase regulator associated with the focal-adhesion-kinase (GRAF), inactivating RhoA and cytoskeletal rearrangement as well as the epithelial re-differentiation of cells, subsequently leading to dormancy [88].

### 4.2. Immune-Mediated and Angiogenic Dormancy

The cancer immunoediting theory refers to a theory encompassing three stages of immune regulation, in relation to cancer progression, proposed and reviewed by Dunn and colleagues [110]. The theory embodies the transition from immune clearance of tumours to steady state equilibrium (immune-mediated dormancy) and ultimately immune evasion and metastatic outgrowth [110]. In this section of the review, immune cells will be discussed in relation to their contributions to the cytokine-regulated, immune-mediated dormancy of breast cancer cells in the bone—with a particular focus on macrophage polarisation.

Within the PMN, macrophages support cytokine-dependent recruitment of fibroblasts, MSCs and osteoprogenitors, as well as promoting the proliferation and differentiation of bone-resident cells and ECM remodelling [27]. More specifically, macrophages have been shown to regulate osteoblast cell function [111], dampen inflammatory cytokine signals during cancer cell seeding, and regulate ECM remodelling proteins within the niche [66]. Collectively, these functions support the recruitment of cancer cells to the niche, as well as PMN formation/maintenance [27,66,111].

**Figure 3:**
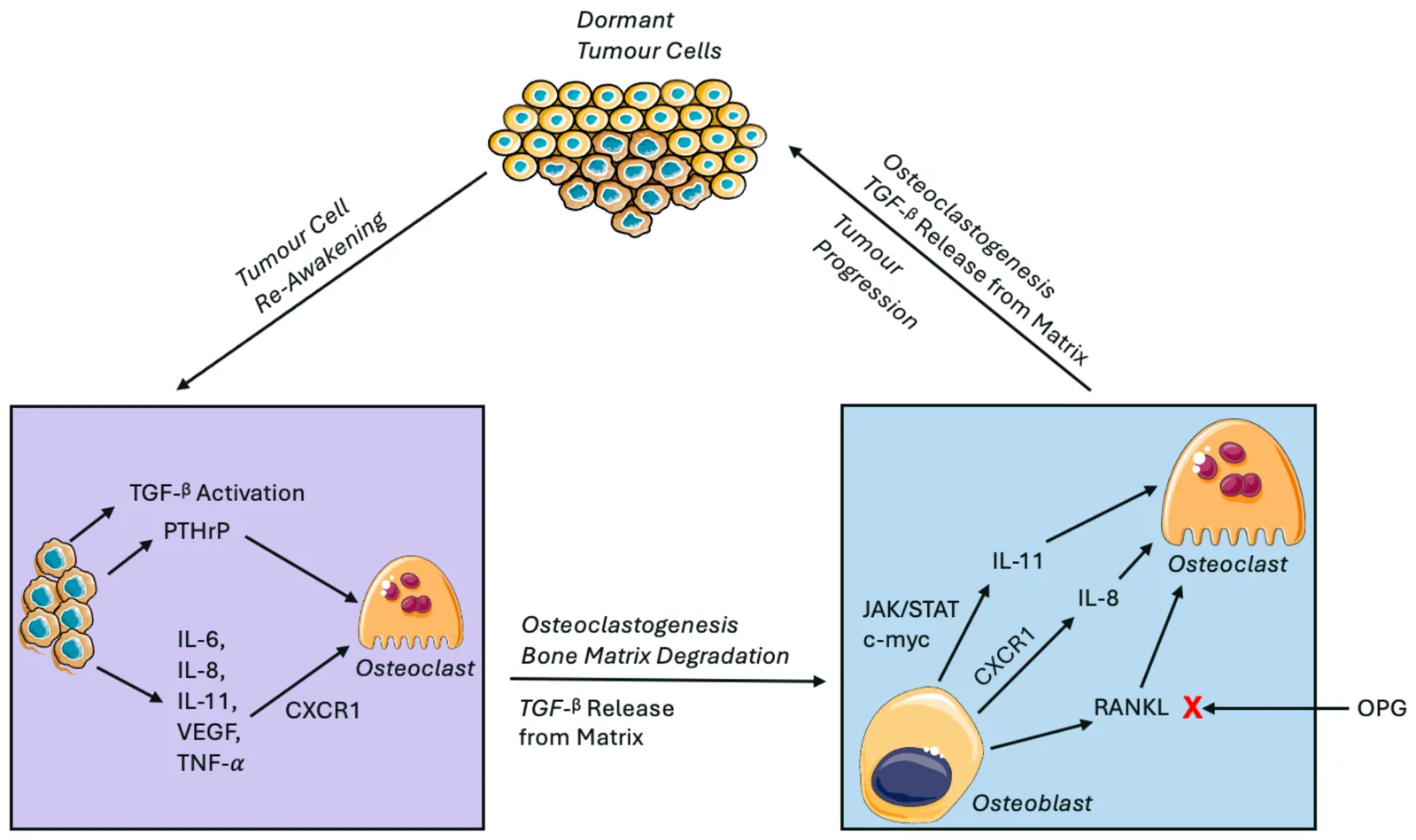
The vicious cycle of breast cancer bone metastasis.

M1-like macrophages are typically induced via IFN-γ, TNF and LPS secretions. They secrete pro-inflammatory cytokines: interleukins 1,2,6 and 12, as well as TNF and the chemokines CXCL9,10 and 11, typically promoting anti-tumour and Th1-driven immunity (Figure 3) [96,97]. In contrast, M2-like macrophages are involved in Th2-immunity, maintaining dormancy in breast cancer bone marrow metastasis through the promotion of type I inflammation and a Th2-driven immune-regulated environment [62,97,112,113]. This is supported by studies on macrophage-derived exosomes in the bone stroma of breast cancer models [62]. Walker et al. determined that naive bone marrow exhibited greater levels of exosomes from M2-like macrophages. Furthermore, immunohistochemical staining of BALB/c femurs, following dormancy reversal induced through anti-miR-222/-223 treatment, uncovered the presence of M2-like macrophages during breast cancer dormancy and M1-like macrophages during dormancy reversal [62]. This was reinforced through the treatment of dormant MDA-MB-231 breast cancer cells with M1-like exosomes, resulting in upregulation of the NFκB subunit, p65, supporting reactivation of breast cancer cell cycling [62]. This is important as it highlights the role of macrophages, and their phenotypes, in relation to the dormancy status of breast cancers in the bone.

The vicious cycle of breast cancer bone metastasis refers to a cytokine-mediated positive feedback loop that drives the metastatic outgrowth of breast cancer cells in the bone, upon dormant cell re-awakening [22]. Cancer cell secretion of parathyroid hormone-related protein (PTHrP), interleukin (IL) 6, 8, 11, vascular endothelial growth factor (VEGF), and tumour necrosis factor alpha (TNF) amplifies RANKL secretion from osteoblasts, upon stimulation by extracellular matrix-released tumour growth factor beta (TGF-β) [99,100]. Independent of RANKL, IL-11 is suggested to drive osteoclast-mediated bone resorption via Janus kinase (JAK)/STAT3, and c-myc and IL-8 via the CXCR1 receptor [114,115,116]. Cumulatively, this amplifies tumour growth as well as further bone degradation, TGF-β release, and repetition of the cycle [99]. Osteoprotegerin (OPG) functions as a decoy receptor for RANKL, therefore intercepting the vicious cycle and hindering osteoclastogenesis as a result [102]. Image(s) provided by Servier Medical Art (https://smart.servier.com), licensed under CC BY 4.0 (https://creativecommons.org/licenses/by/4.0/) (Accessed on 23 August 2026).

In contrast, additional studies have demonstrated M2-skewed macrophages to cluster at vascularised regions in 4T1 and MMTV-PyMT orthotopic breast cancer models. This was specifically following chemotherapy treatment, and suggested to promote tumour relapse following therapy [113]. Cumulatively, these findings reinforce the dual role of tumour-associated macrophages (TAMs) regarding dormancy and reactivation, but also the need for future experiments to validate their role in the bone specifically.

Inflammatory monocytes and MDSCs have also been found to contribute to cancer cell dormancy within the niche [94]. Upon cancer cell arrival, inflammatory monocytes are suggested to increase their trafficking to the niche, as well as recruit MDSCs [94]. Chen et al. demonstrated that breast cancer cell-derived VEGF-c induces chemokine cascades that lead to lymph node MDSC recruitment and the suppression of anti-tumour immunity, highlighting the immune suppressive characteristics of MDSCs in breast cancer [95]. Furthermore, dormant cancer cells have also been identified to secrete IL-10 and TGF-β to downregulate T and NK cell activity in the bone, sustaining immune evasion during dormancy [91]. However, it is important to note that although immune evasion is permissive for dormancy, it also supports outgrowth/metastasis of cancer upon appropriate environmental changes—immunoediting theory [117].

Regarding the role of angiogenesis during breast cancer dormancy in the bone, hypoxia is known to support metastatic outgrowth, partially through stimulation of angiogenesis and secretion of pro-angiogenic factors [118]. In line with this, CXCL9 has been identified as an anti-angiogenic chemokine and is associated with the dormancy of cancers [84]. In breast cancer patients, dormant disseminated tumour cells (DDTCs) were found in the bone marrow alongside elevations in CD4^+^ and CD8^+^ T cells [119]. Interestingly, CXCL9 has been associated with CD4^+^ and CD8^+^cell recruitment, and in a pancreatic cancer model, CD4^+^ Th1 cell IFN-γ secretion and TNFR1 expression promoted growth arrest and dormancy of cancer cells [84,120]. Although the above model is not breast cancer specific, osteoblasts also secrete CXCL9 in the bone and its implications in CD4^+^/ CD8^+^ cell recruitment in breast cancer dormancy could be explored further in the future. Moreover, CXCL9 has been associated with the inhibition of angiogenesis via blocking VEGF binding to endothelial cells. This is important as angiogenesis has been previously shown to promote the invasion and proliferation of dormant breast cancer cells in a post-menopausal obese mouse model [84,89,90]. Collectively, with the need for future and additional experimental validation, the above findings highlight the implications of CXCL9-mediated angiogenic and immunologic dormancy of breast cancers disseminated to the bone.

In contrast to CXCL9, CXCL10 was previously understood to have pro-tumorigenic functions [85]. However, preliminary findings from The University of Bristol unravelled the role of CXCL10 and its receptor CXCR3 in maintaining the immune-mediated dormancy of breast cancers [86]. Researchers found that CXCL10 suppression reduced T and B cell infiltration into the tumour microenvironment and increased the burden of tumour cells in the lung metastatic niche [86]. Future experiments may show similar effects in bone metastasis; however, research into the CXCL9/10/CXCR3 axis in breast cancer dormancy is newly emerging and therefore further work to validate these findings and their underlying mechanisms is necessary [86].

## 5. Metastatic Outgrowth of Breast Cancers in Bone

As outlined above, osteoblasts are the source of a variety of pro-dormancy cytokines. However, osteoblasts are also known to secrete IL-6, MCP-1, VEGF, macrophage inflammatory protein 2 (MIP-2), CXCL12, BMP, and Notch to sustain cancer cell growth and recruitment to the niche (Figure 3) [3]. It is not yet fully understood what causes osteoblasts and other bone marrow-derived cells to transition from the secretion of pro-dormancy to pro-tumour factors. However, it has been suggested that this switch may be the result of changes to the local microenvironment and potentially induced by stress, ageing, hormone receptor expression and trauma, including trauma-associated inflammation [121].

Moreover, immune cells play a pivotal role in creating a supportive local environment for the awakening and proliferation of dormant cancer cells in the bone [97], and the role of cytokines, in the context of tumour outgrowth, will be discussed in the following sub-sections.

### Cytokine-Regulated Outgrowth of Breast Cancers in the Bone: Osteoblast and Immune Cell-Derived

IL-1β is hypothesised to serve as a key driver of DDTC re-awakening in the bone. Patients with high IL-1β expression in their primary tumours show increased risk of recurrence in the bone [3,33]. In addition, pharmacological inhibition of IL-1β signalling prevents metastatic outgrowth of disseminated cancer cells present in the bone niche of mice [98], and bone metastases do not form from disseminated cancer cells in the bones of *IL-1β* knockout mice [61]. Importantly, although inhibiting IL-1β prevents the development of overt metastases, inhibiting this cytokine does not alter the number of disseminated cancer cells in mouse bone, but maintains these cells in a dormant state [7,98]. These findings support IL-1β as a potent driver of tumour outgrowth in the bone of breast cancer patients [7,98].

The mechanism by which IL-1β promotes metastatic outgrowth appears to be multi-faceted: IL-1β in the bone microenvironment has been shown to stimulate outgrowth of dormant tumour cells via the activation of Wnt signalling in the tumour cells [7]. In addition, co-culture studies between bone osteoblasts, or HSCs, and MDA-MB-231/T47D breast cancer cells revealed amplified IL-1β following tumour cell–niche interaction, increasing the proliferation of both niche and cancer cell subtypes [33]. However, direct addition of IL-1β to niche cells or tumour cells resulted in increased proliferation of osteoblasts and HSCs, but not the cancer cells [33]. This observation suggests that bone and cancer cell interaction within the niche stimulates IL-1β secretion as a mechanism of niche expansion, supporting cancer cell exit from dormancy [33]. Furthermore, unlike at other sites, IL-1β has been shown to reduce anti-cancer immunity in bone via reducing the infiltration of myeloid cells and neutrophils [61].

In addition to IL-1β, IL-6 has been found to be secreted by osteoblasts when in contact with breast cancer cells [122]. IL-6 can stimulate osteoblasts to secrete higher levels of RANKL and drive osteolysis, feeding into the vicious cycle of bone metastasis (Figure 3) [22,123]. The vicious cycle includes cancer cell secretion of parathyroid hormone-related protein (PTHrP), IL-6, 8 and IL-11, VEGF, and TNF upon stimulation by TGF-β during ECM remodelling. As a result, osteoblast-derived RANKL secretion is exacerbated, as well as the cycle itself, supporting outgrowth of breast cancers in the bone [22,99,100]. Furthermore, osteoblast-derived RANKL is able to stimulate breast cancer cells to secrete further IL-6 [123]. IL-6 then upregulates RANK expression on the surface of cancer cells, and once again, secretion of IL-6 as a positive feedback loop [123]. Increased RANKL and RANK expression will drive RANK-dependent osteoclastogenesis, bone resorption and further TGF-β release. Once again, positively reinforcing the cycle [22,99,100,122,123].

Independent of RANKL, or its secretion from cancer cells directly, IL-11 is also secreted by osteoblasts following breast cancer stimulation/interaction in the bone [100,124]. Osteoblastic secretion of IL-11 is cyclooxygenase (COX-2) or TGF-β mediated, and its overexpression not only induces osteolytic effects but also angiogenic effects [124]. Initial findings suggest that cancer-driven activation of TGF-β stimulates the release of IL-11 from osteoblast cells in the local environment, and therefore osteolysis [114]. This mechanism is suggested to be driven by JAK/STAT3 signalling of c-Myc-driven osteoclastogenesis [115]. Similarly, IL-8 has also been associated with increased osteoclastogenesis; however, via both RANKL-dependent and independent mechanisms. IL-8 directly increases osteoclastogenesis via the CXCR1 receptor on the surface of osteoclasts, or indirectly via increasing osteoblast lineage-derived RANKL secretion [116,122], feeding into the above vicious cycle of breast cancer bone metastasis.

Importantly, in addition to osteoblast-secreted interleukins, immune cell secretion of interleukins has also been found to contribute to breast cancer dormancy/outgrowth in the bone. For example, IL-17A secreted by Th17 cells has been found to activate secretion of matrix metalloproteinases (MMPs) and VEGF, via an IL-17A/IL-6/STAT3 cascade [103]. MMPs are a family of proteinases that play key roles in ECM degradation and cytokine processing, and although not cytokines themselves, have been associated with the TGF-β-driven growth of breast cancers within the bone [104].

Interestingly, during CD4^+^ Th17 differentiation, TAMs are known to secrete CXCL12 and CCL2 [103], as well as macrophage and breast cancer cells secreting IL-6 or TGF-β [103]. IL-17A secreted by the Th17 cell type further stimulates IL-6, 8 and 1β secretion in the TME via the MAPK/p38 pathway, highlighting IL-17A involvement in the vicious cycle of breast cancer metastasis, but also potential CXCL12-mediated recruitment of circulating cancer cells during Th17 differentiation [103]. In addition, IL-17A-dependent IL-6 and MMP secretion has previously been shown to play a role in bone resorption [125]. More specifically, high IL-17A was found in autoimmune arthritis, and upon injecting the same arthritic mouse model (high IL-17A) with 4T1 breast cancer cells, revealed increased bone metastases, demonstrating a role for IL-17A in the metastatic outgrowth of bone disseminated breast cancers [125].

Similarly, IL-22 was found to be elevated in aggressive and metastatic breast cancers, as well as driving bone invasion of breast cancer cells via MMP-9 expression and sphingosine-1-phosphate (S1P) signalling [105]. In this system, leukocyte-secreted MMP-9 activates TGF-β and LOX, creating an environment that supports the outgrowth of dormant cancer cells [106]. Previous studies in hepatocellular carcinoma models displayed leukocyte infiltration in the presence of IL-22 overproduction [105]. In response to this, Kim et al. uncovered IL-22 signalling to drive leukocyte recruitment, specifically macrophage accumulation, in a bone marrow MSC-derived MCP-1-dependent manner [105].

As well as MMP-9, MMP-2 has also been associated with cancer aggressiveness and invasiveness, through stimulating degradation of the ECM [104]. Intra-cardiac injection of MMP-2 overexpressing MDA-MB-231 breast cancer cells into mouse models has demonstrated increased bone metastases [126]. However, tumour-derived MMP-2 and MMP-2 derived from the microenvironment may have differential effects on metastatic outgrowth. It has been suggested that ECM fragments, released via MMP-2-driven degradation, could inhibit angiogenesis and promote breast cancer dormancy in the bone niche [104]. In contrast, genetic knockout of *MMP-2* in mouse models leads to reduced growth of breast cancer bone metastases, with reduced levels of TGF-β detected in the system [104]. Thiolloy et al. reinforced the role of MMP-2 in breast cancer bone metastasis, specifically in relation to its ability to regulate TGF-β bioavailability [127]. Using *MMP-2* null/wild type mouse models, the group uncovered osteoblast- and host-derived MMP-2 to be critical for breast cancer cell survival in the bone metastatic niche [127]. Osteoblast-derived MMP-2, specifically, was found to promote the activation of TGF-β following its liberation from the matrix [127].

Downstream from TGFβ and PTHrP activation, the importance of RANKL is demonstrated through use of a pharmacologically enhanced OPG (OPG-Fc) [101]. OPG is a decoy receptor for RANKL, and therefore hinders RANK/RANKL downstream signalling and osteoclastogenesis (Figure 1 and Figure 3) [102]. Importantly, RANKL-driven osteoclast activation is shown to reactivate dormant cells in the endosteal niche [128], and RANKL secretion from osteoblasts is exacerbated by the presence of cancer cells in the niche [101]. Importantly, OPG-Fc administration overrode RANK–RANKL interactions and inhibited the outgrowth of dormant breast cancer cells [101]. This increase in RANK was associated with increased osteolytic bone metastasis in an MDA-MB-231 breast cancer model [101].

In addition to the above cytokines, chemokines have demonstrated a crucial role in the outgrowth of tumours within the bone. The chemokine CXCL5 is secreted by both cancer and bone marrow cells and, by binding to its receptor CXCR2, regulates angiogenesis, migration and invasion of tumours [107]. Importantly, cultures of cancer-bearing bone (bone explant cultures from mice injected intra-cardiac with PyMT cells) were found to secrete more CXCL5 in comparison to the ex vivo healthy bone explant cultures [107]. Both CXCL5 and CXCR2 have also been found in bone tissue from breast cancer patients [107], potentially implicating this signalling pathway in the metastasis of breast cancers to the bone. However, further work is necessary to validate this.

In line with the above, CCL5 has also been associated with the outgrowth of breast cancers and dormancy exit in the bone niche [108]. In breast cancer, CCL5 is secreted into the TME by MSCs, with high levels of CCL5 associated with the promotion of cancer recurrence in patients [108]. Furthermore, CCL5 expression by the primary tumour is involved in TAM recruitment [108] and, in breast cancer specifically, these TAMs were associated with the expression of VEGF, M2-like phenotype, and immune evasion, and hence, poor prognosis [108,129]. It could be hypothesised that the same observations would apply for the bone metastasis of these breast tumours, but further work would be necessary to explore this.

Conversely, VEGF has been hypothesised to play a role in tumour exit from dormancy, but due to its angiogenic functions [130]. VEGF is suggested to experience increases in its expression due to hypoxia and the subsequent stimulation of hypoxia-inducible factors (HIFs) [131]. Importantly, additional studies have also uncovered the importance of niche expansion in the re-awakening of dormant cancer cells residing in the bone [33]. Angiogenesis, stimulated by VEGF, is said to contribute to niche expansion and the progression of dormant micro-metastases into overt metastatic lesions [33]. Inhibition of IL1β (IL-1 receptor KO mouse models) resulted in reduced VEGF and endothelin 1 concentration in the bone marrow of mice, as well as reduced vessel length (CD34^+^) [33]. Furthermore, immune cells are a key source of VEGF and IL-1β, further driving immune infiltration of the TME [3,131]. This highlights the positive feedback between VEGF, the IL family and the immune milieu of the bone, but also reinforces the inter-linking relationships between various cytokine families, the immune system, and bone in relation to the regulation of skeletal tumour dormancy and outgrowth.

## 6. Integrative Perspective

Several cytokines discussed in this review, including TGF-β, IFN-γ, VEGF and CXCL12, perform roles across pre-metastatic niche priming, dormancy and outgrowth of disseminating breast cancers. Little is known about the specific causes of dormancy-outgrowth switching in breast cancer; however, stress, inflammation and age are suggested to play a role [121], with various factors contributing to the multi-faceted role of these cytokines in dormancy or outgrowth of breast cancers.

For example, TGF-β has been discussed with regard to its dual role in breast cancer progression [132]. In breast cancer bone metastasis, specifically, Smad signalling was uncovered to regulate the pro- or anti-tumour functions of TGF-β [132]. During the early stages of tumorigenesis, TGF-β suppresses tumour growth; however, it later undergoes a shift in functionality to favour the metastatic progression of the cancer. In a model of breast cancer bone metastasis, MDA-MB-231 breast cancer cells silenced for either Smad2 or 3 were injected into the left cardiac ventricle of mice. Interestingly, Smad2-deficient cells expressed a statistically significant (*p* < 0.001) aggressive phenotype of breast cancer, in comparison to the Smad3-deficient cells, which displayed increased time for tumour growth [132]. Furthermore, in Smad2-depleted models, three-fold greater VEGF expression was reported [132]. It may be argued that during the early stages of bone colonisation, TGF-β-Smad2 signalling maintains tumour cells in a latent phase and upon cancer cell–niche cell interaction and hyper-secretion of TGF-β via the vicious cycle of bone metastasis, Smad3-dependent signalling is favoured, mediating VEGF release [22,132]. This is important as rapid increases in VEGF have been previously reported to cause an angiogenic switch, supporting dormancy reversal in breast cancer [133]. Furthermore, in addition to its role in breast cancer bone homing and dormancy, the CXCL12–CXCR4 axis has been found to increase VEGF expression via Akt signalling [66,80,134]. This was measured by RT-PCR in vitro and confirmed through an MDA-MB-231 breast cancer in vivo mouse model angiogenesis assay [134]. This supports the idea of the osteogenic–angiogenic switch promoting breast tumour growth/exit from dormancy, but also highlights the cytokine crosstalks regulating breast cancer dormancy in the bone, and the multi-faceted nature of their individual functions.

Similar to TGF-β, IFN-γ is a pleotropic cytokine that regulates dormancy, immune evasion, and escape of dormant breast cancer cells through immune-mediated mechanisms [135,136]. MCF-7 and MDA-MB-231 breast cancer cells, treated with IFN-γ for 24 h, underwent cDNA microarray analysis followed by gene ontology, STRING and Oncomine gene expression analysis to identify IFN-γ regulated genes in breast cancer [135]. The results displayed genes induced by IFN-γ to play a role in cell-cycle progression, e.g., *DYNLL1* and *MAD1L1*. Furthermore, genes identified to be repressed by IFN-γ were involved in breast cancer progression: *KIF20B*, *RALA*, *PSMD8* and *AURKA.* Interestingly, it was observed that at 24 h, treatment with IFN-γ led to morphological changes in both breast cancer cell lines, and these changes were associated with both increased migration and also the inhibition of growth and invasion, highlighting the multi-faceted role of IFN-γ [135]. Importantly, over this time course, it was also reported that the breast cancer cells experienced reduced expression of the IFNGR1 receptor. It was hypothesised that the chronic exposure to IFN-γ by NK and T cells resulted in adaptation of the mammary tumour to repress receptor expression [135]. These observations are reinforced by studies conducted in FVB mice, whereby high levels of IFN-γRa expression on mouse mammary carcinoma cells resulted in CD8^+^ T cell-mediated relapse-free survival, suggested to mimic tumour cell dormancy, but low levels facilitated tumour relapse [136].

This highlights the crosstalk between cytokine networks regarding dormant cell fate in the bone niche, as well as the complexity in targeting dormancy mechanisms for therapeutic intervention, owing to the diverging roles of these cytokines. However, most of all, this highlights the need for a deeper understanding of these mechanisms to uncover novel interventions.

## 7. Current Therapeutic Approaches

In the bone, dormant breast cancer cells persist during and after treatment of the patients’ primary tumour, exhibiting resistance mechanisms in response to standard chemotherapy regimens [137]. In addition to dissemination taking place early and dormancy impeding our ability to prevent metastatic spread, this makes dormant cells difficult to target and eliminate [5,6,7].

Cumulatively, therapeutic interventions for breast cancer recurrence must target dormant cells within the bone to either maintain their dormancy, target them in a dormant state or target them upon reactivation [137]. Targeting disseminated breast cancer cells upon their reactivation poses high risks, whereby, in the case of treatment failure, the cancer may progress into an overt metastatic lesion which would, at this stage, be therapy resistant [137]. Hence, it may be argued that targeting the disseminated cells during dormancy is the most viable therapeutic intervention. Current approaches, although limited, range from exploring epigenetic targets and the utilisation of targeted approaches such as CDK4/6 inhibition or endocrine-targeted approaches, as reviewed in [137]. To date, there has been one clinical trial specifically aiming to determine the effects of long-term Tamoxifen on late-onset metastasis: the ATLAS randomised trial explored the administration of Tamoxifen for up to 10 years after diagnosis of oestrogen receptor positive (ER+) breast cancer [138]. However, this trial reported a recurrence rate of 18% in patients treated for 10 years compared to 20.8% in patients treated for up to 5 years [138]. Although a step in the right direction, improvements in patient outcome were low and this regimen did not prevent re-awakening of dormant tumour cells.

Therapies corresponding to the specific cytokines discussed in this review include those targeting IL-1β, IL-6, TGF-β, RANKL, and CXCR4. Preclinical studies exploring the monoclonal antibody inhibition of IL-1β (Canakinumab) or IL1R1 receptor antagonist Anakinra have uncovered the ability for these drugs to stop metastasis of breast cancers as well as their outgrowth in the bone—although neither of these biologics is currently approved for their application in oncology [139]. Interestingly, combining Anakinra with Doxorubicin/Zoledronic acid reduced distal recurrence in the bone and reduced growth of the primary tumour, suggesting potential for combining IL-1 targeted treatments with standard of care in future clinical trials [61].

Additional preclinical findings include targeting IL-6 via a neutralising antibody. This inhibition of IL-6 was found to significantly reduce dormancy escape of breast cancers when explored in an orthotopic BALB/c dormant (D2.0R) breast cancer model, and in response to taxane chemotherapy [140]. Neutralising antibody treatment has also been explored in preclinical studies targeting TGF-β, whereby inhibition of TGF-β in orthotopic (MDA-MB-231/4T1) breast cancer mouse models was found to reduce metastasis, but also improve response to doxorubicin [141].

Whilst targeting the RANKL/OPG axis in mouse models has been shown to maintain tumour cells in a dormant state, hindering metastatic outgrowth of breast tumours [101], clinical trials using Denosumab, a monoclonal antibody treatment that targets RANKL [142], have demonstrated reduced osteoclastic maturation and activity but have no effect on bone metastasis [143]. These data suggest that inhibition of RANKL/OPG signalling may be insufficient to retain cells in a dormant state in humans, which poses questions around the relevance of mouse models for studying human disease mechanisms.

Lastly, targeting of the CXCL12-CXCR4 axis has been explored pre-clinically to prevent tumour cells from lodging within the niche. Administration of the CXCR4 antagonist, AMD3100, to patient-derived xenograft models resulted in impaired lung metastasis in HER2+ breast cancer but not triple negative breast cancer [144]. However, when used in combination with ionising radiation, AMD3100 treatment has been reported to increase cell-cycle arrest and apoptosis in triple negative breast cancer, but this study did not look at regulation of dormancy or effects on dormant cancer cells [145]. With regard to bone metastasis, mixed results have been reported; in mouse models of ER+ and triple-negative bone metastasis, administration of AMD3100 mobilises haematopoietic stem cells from the niche into the circulation, resulting in increased numbers of tumour cells being disseminated into trabecular bone. Therefore, rather than inhibiting tumour cell homing to bone, this drug appears to increase bone homing [146]. In contrast, simultaneous targeting of the TRV6 calcium channel and CXCR4 has been shown to prevent bone metastasis in mouse models of castrate-resistant prostate cancer [147]. It must be noted that all of these models rely on a mouse bone environment and further experimental validation is required [5,6,7], reinforcing the need to develop more clinically relevant models for the successful development of novel therapeutic strategies for late-onset metastasis.

## 8. Summary/Conclusions

Various families of cytokines play key roles in both the dissemination and homing of breast cancers to the bone pre-metastatic niche, as well as their subsequent dormancy and outgrowth. Importantly, cytokines support homeostasis of the bone microenvironment, and those responsible belong mainly to the TNF, IFN and IL families, as well as RANKL. Dysregulation of the secretion of these cytokines has been associated with abnormal bone degradation and formation impacting cancer cell seeding, quiescence or active proliferation at specific metastatic niches within bone. The predominant chemokines and cytokines involved in the dormancy of breast cancers at the pre-metastatic niche include CXCL12, LIF, CXCL9/10, TGF-β, BMP, FGF, OPG, and various IL family members, as well as macrophage polarisation and immune cell infiltration being identified as a key source of cytokines for the niche. Cytokines identified to drive the transition from quiescence to outgrowth, in the metastatic niche, include the IL family, TGF-β, PTHrP, VEGF and chemokines CXCR4, CXCL5 and CCL5. This review highlights the holistic nature of cytokine regulation in the bone niches, regarding breast cancer dormancy and outgrowth, encompassing the importance of immune signalling and inflammation, with regard to breast cancer progression into overt metastatic lesions. Our findings not only highlight the requirement for further research in this area and the development of novel therapeutic interventions, but also the potential of various cytokines and immune networks to serve as preventative targets in the distal recurrence of breast cancer in the bone.

## Figures and Tables

**Table 1:** Cytokine and immune cell regulation of breast cancer dormancy and outgrowth in the bone pre- and metastatic niches.

Dormancy	Outgrowth
LIF	TNF
CXCL9/10	CXCL9, 10, 11
TGF-β	TGF-β
FGF-2	IL-1, 2, 6, 12
VEGF	VEGF
IL-10	IL-8, 11
OPG	PTHrP
	RANKL
	IL-17A/IL-22
	LOX
	CXCL5/CXCR2
	CCL5
	IL-1β

## Data Availability

No new data were created or analyzed in this study. Data sharing is not applicable to this article.
